# Novel Human Meningioma Organoids Recapitulate the Aggressiveness of the Initiating Cell Subpopulations Identified by ScRNA‐Seq

**DOI:** 10.1002/advs.202205525

**Published:** 2023-03-30

**Authors:** Meng Huang, Shao Xu, Yuzhe Li, Li Shang, Xiudan Zhan, Chaoyin Qin, Jun Su, Zijin Zhao, Yi He, Lina Qin, Wei Zhao, Wenyong Long, Qing Liu

**Affiliations:** ^1^ Key Laboratory of Stem Cells and Tissue Engineering Sun Yat‐Sen University Ministry of Education 510080 Guangzhou China; ^2^ Medical Research Institute Guangdong Provincial People's Hospital Guangdong Academy of Medical Sciences Southern Medical University 510080 Guangzhou China; ^3^ Guangdong Cardiovascular Institute Guangdong Provincial People's Hospital Guangdong Academy of Medical Sciences 510080 Guangzhou China; ^4^ Department of Neurosurgery in Xiangya Hospital Central South University 410008 Changsha China; ^5^ Department of Pathology in Xiangya Hospital Central South University Changsha 410008 China; ^6^ Department of Neurosurgery Hunan Children's Hospital Changsha 410007 China

**Keywords:** cell subpopulation, meningioma, patient‐derived organoid model, single‐cell RNA sequencing

## Abstract

High‐grade meningioma has an unsatisfactory outcome despite surgery and postoperative radiotherapy; however, the factors driving its malignancy and recurrence remain largely unknown, which limits the development of systemic treatments. Single‐cell RNA sequencing (scRNA‐Seq) technology is a powerful tool for studying intratumoral cellular heterogeneity and revealing the roles of various cell types in oncogenesis. In this study, scRNA‐Seq is used to identify a unique initiating cell subpopulation (SULT1E1^+^) in high‐grade meningiomas. This subpopulation modulates the polarization of M2‐type macrophages and promotes meningioma progression and recurrence. A novel patient‐derived meningioma organoid (MO) model is established to characterize this unique subpopulation. The resulting MOs fully retain the aggressiveness of SULT1E1^+^ and exhibit invasiveness in the brain after orthotopic transplantation. By targeting SULT1E1^+^ in MOs, the synthetic compound SRT1720 is identified as a potential agent for systemic treatment and radiation sensitization. These findings shed light on the mechanism underlying the malignancy of high‐grade meningiomas and provide a novel therapeutic target for refractory high‐grade meningioma.

## Graphical abstract

SULT1E1^+^ cell subpopulation may directly modulate the behavior of other tumor cells and trigger the M2‐like polarization of macrophages in high‐grade meningiomas. The novel patient‐derived meningioma organoids fully retain the intratumoral cellular heterogeneity and demonstrate invasiveness after orthotopic transplantation. SRT1720 is a potential agent for systemic treatment and radiation sensitization for high‐grade meningiomas by inhibiting SULT1E1^+^ cell subpopulation.

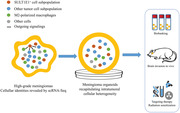

## 1 Introduction

Meningiomas are the most common primary intracranial tumors worldwide, accounting for 38.3% of the central nervous system neoplasms.^[^ ^1^ ^]^ Arising from the arachnoid cap cells of the meninges, ≈80% of meningiomas are classified as benign (grade I) based on the mitotic rate, and surgical removal is the only treatment necessary for most of these patients.^[^ ^2^ ^]^ However, for high‐grade meningiomas (grades II and III), a high rate of recurrence and increased mortality are observed despite surgical resection and routine postoperative radiotherapy.^[^ ^3^ ^]^ A previous multi‐center retrospective analysis of 199 patients with high‐grade meningiomas showed that the 10 year progression‐free survival rates for grade II and III meningiomas were 22.6% and 0%, respectively.^[^ ^4^ ^]^ These poor outcomes indicate the inadequacy of the current treatment strategy; thus, it is crucial to determine the effective molecular target required to improve treatment for this group of patients in the future.

Recently developed sequencing technologies (“next‐generation sequencing”) have revealed the genomic and epigenetic landscapes of meningiomas.^[^ ^5^ ^]^ The identification of genetic alterations such as NF2, AKT1, TRAF7, SMO, and PIK3CA offers insight into the molecular mechanisms of tumorigenesis in meningiomas. Nonetheless, a poor understanding of the key factors that drive the malignancy of grade II and III meningiomas has hindered the development of effective drugs. Although TERT promoter mutations and homozygous deletion of CDKN2A/B have been reported to be associated with high aggressiveness and early recurrence in meningioma,^[^ ^6^ ^]^ only a small population of patients with malignant meningioma harbor these genomic alterations. Therefore, it is crucial to elucidate the essential factors that distinguish high‐grade meningiomas from benign meningiomas to promote the development of novel therapies.

Intratumoral heterogeneity plays an important role in the initiation, evolution and progression of aggressive malignancies.^[^ ^7^ ^]^ For high‐grade meningiomas, intratumor heterogeneity has also been highlighted in many aspects, such as transcriptomics, epigenomics and DNA methylation profiles.^[^ ^8^ ^]^ However, this feature has not yet been illustrated from the perspective of cellular identity. Single‐cell RNA sequencing (scRNA‐Seq) is a powerful tool for studying intratumoral cellular heterogeneity and identifying different cell subpopulations with unique gene expressions and functions.^[^ ^9^ ^]^ In this study, we performed a scRNA‐Seq analysis of clinical meningioma samples and identified a unique subpopulation of meningioma tumor cells. We identified this subpopulation as a key factor in controlling the aggressiveness of high‐grade meningiomas. Next, we developed a novel patient‐derived meningioma organoid (MO) model in which we were able to reproduce the heterogeneity of meningiomas. This milestone enabled us to investigate this unique cellular subpopulation with the aim of developing effective therapy. These results provide new insights into the driving factors of high‐grade meningiomas and a potential therapeutic target.

## 2 Results

### 2.1 M2‐Like Polarization of Macrophages in High‐Grade Meningiomas

**Figure 1:**
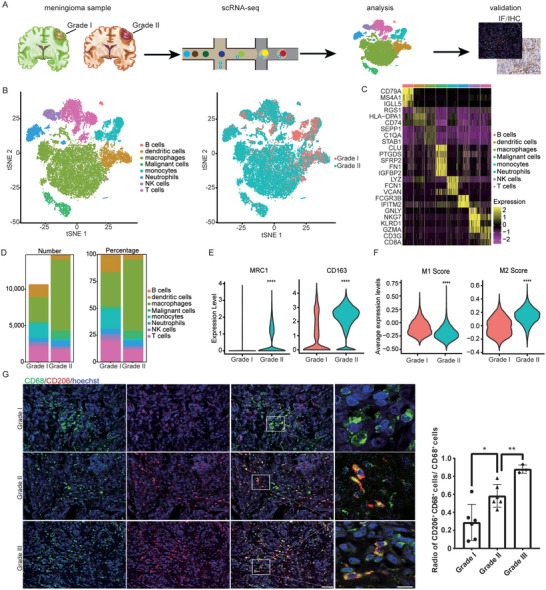
scRNA‐Seq confirms enhanced M2‐like polarization in tumor‐associated macrophages (TAMs) in grade II/III meningiomas. A) Schematic representation of the experimental strategy. B) *t*‐distributed stochastic neighbor embedding (*t*‐SNE) plots of cell clusters across tumors, colored by cell type (left) and the World Health Organization (WHO) grade of the original tumors (right). C) Heatmap showing signature genes expressed in the indicated cell types. D) Histogram indicating the number and proportion of cells in tumor tissue of grades I and II. E) Violin plots of the expression of M2 phenotype‐relevant genes in clusters of macrophages and monocytes. F) Calculation of M1 and M2 scores indicating M2‐like macrophage polarization in grade II meningioma sample. G) Representative images and quantification of immunostaining with anti‐CD68 and anti‐CD206 antibodies, confirming the M2‐like polarization of TAMs in grade II and III meningiomas. Scale bars: 20 µm (left), 10 µm (right).

To investigate the differences in cell populations and molecular characteristics between grade I and grade II/III meningiomas, 25 617 cells were isolated from primary tumors of two grade I and one grade II meningioma sample. scRNA‐Seq was performed using the 10× Genomics Chromium platform, and data originating from these cells were included for analysis and validation (**Figure** **1**A). Using the “Seurat” R package, we applied *t*‐distributed stochastic neighbor embedding (t‐SNE) and visualized the cell‐type results. Overall, eight cell clusters were identified, including tumor‐infiltrating B lymphocytes, dendritic cells, macrophages, monocytes, neutrophils, NK cells, T cells, and malignant cells (Figure 1B). We next investigated differential gene expression to identify genes expressed exclusively in each cell cluster (Figure 1C). Many of these were well‐known cell markers (Figure S1A, Supporting Information). Gene ontology (GO) enrichment analysis confirmed that these exclusively expressed genes were enriched in particular pathways of the corresponding cell types (Figure S1B, Supporting Information). Notably, in grade II meningioma, the macrophage population showed a greater cell number and proportion than in grade I meningiomas (Figure 1D). We further evaluated the M1/M2‐like polarization status of the macrophage/monocyte population. The results showed that the expression of the M2 phenotype‐related genes MRC1 and CD163 was markedly higher in grade II meningioma (Figure 1E and Figure S1C, Supporting Information). Moreover, compared with grade I, grade II meningioma sample showed significantly lower M1 score and higher M2 score (Figure 1F and Figure S1D, Supporting Information). Immunofluorescence staining for the M2 macrophage marker CD206 and the monocyte/macrophage marker CD68 in formalin‐fixed paraffin‐embedded (FFPE) tissues of meningioma samples (Figure 1G) demonstrated an increased CD206^+^/CD68^+^ cell ratio in grade II/III meningiomas, confirming the M2‐like polarization of macrophages. Taken together, these results confirmed that the M2‐like polarized macrophages are a dominant component of high‐grade meningiomas, which is consistent with the results of a previous study.^[^ ^10^ ^]^

### 2.2 Unique Meningioma Cell Subpopulation in High‐Grade Meningiomas

**Figure 2:**
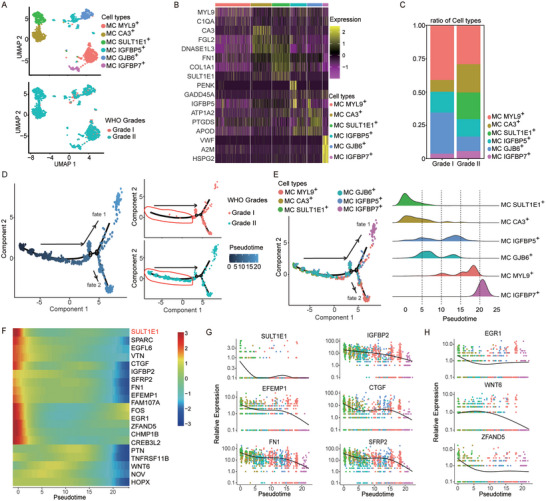
scRNA‐Seq identification of an exclusive meningioma cell subpopulation in the grade II meningioma sample. A) Uniform Manifold Approximation and Projection for Dimension Reduction (UMAP) plots showing different MC subclusters across meningioma tumor samples, colored by cell subcluster (upper) and by the WHO grade of the original tumor (lower). B) A heatmap showing the signature genes expressed in different MC subclusters. C) Quantification of the proportions of the different MC subpopulations in the samples, revealing that the MC SULT1E1^+^ subcluster appears only in grade II meningioma sample. D) A pseudotime trajectory for MCs calculated by the Monocle application (left); tumor grades are labeled by color. E) A Monocle pseudotime trajectory for different MC subpopulations, revealing that the SULT1E1^+^ subcluster is distributed in the beginning of trajectory. F) Heatmap showing the dynamic changes in gene expression along the pseudotime trajectory. G) Expression of the oncogenic genes of the SULT1E1^+^ subcluster on a pseudotime scale, colored by different MC subpopulations. H) Expression of the transcription factor genes of the SULT1E1^+^ subcluster on a pseudotime scale, colored by different MC subpopulations.

Since M2‐like macrophage polarization is widely associated with tumorigenesis, we next sought to determine the contribution of various sub‐clusters of meningioma cells (MCs) to M2‐like polarization. The identity of the tumor cells was further validated by copy‐number variation analysis (Figure S2A, Supporting Information). Graph‐based clustering and Uniform Manifold Approximation and Projection (UMAP) were performed to visualize the sub‐clusters of the MCs. A total of six subpopulations were identified, each of which was named after its signature expressing genes (**Figure** **2**A,B). GO enrichment analysis indicated that these MC subpopulations had different functions in tumor development (Figure S2B, Supporting Information). Notably, the MC SULT1E1^+^ subpopulation was found only in the grade II meningioma sample (Figure S2C, Supporting Information), which was confirmed by quantifying the proportions of the different subpopulations in the meningioma (Figure 2C). We next performed a pseudotime analysis to better understand the function and dynamic changes of these tumor cell subpopulations. The result showed that many tumor cells in grade II meningiomas were at the beginning of the trajectory (Figure 2D). Moreover, distinctive cell functions were observed in the relatively early, middle, and late part in the pseudotime trajectory, showing a functional transition from endothelial development and differentiation to extracellular structure formation (Figure S2D, Supporting Information). When we integrated the cell clustering and pseudotime trajectory data, we found that the MC SULT1E1^+^ subpopulation was predominantly distributed at the beginning of the trajectory, revealing a different differentiation status with other subpopulations (Figure 2E). On a pseudotime scale, the signature genes of MC SULT1E1^+^ were highly expressed at the beginning, and their expression gradually decreased (Figure 2G,H). In summary, we identified a tumor cell subpopulation, MC SULT1E1^+^, which was exclusive to the grade II meningioma sample and might plays an important role in the tumor progression.

### 2.3 Validation and Study of the MC SULT1E1^+^ Subpopulation

**Figure 3:**
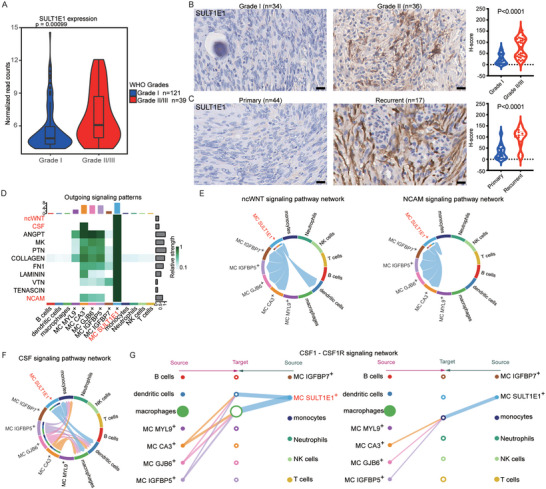
Comprehensive analysis of the function of the MC SULT1E1^+^ subpopulation. A) Violin plot showing the expression differences of SULT1E1 between grade I and grade II/III meningiomas using the Gene Expression Omnibus (GEO) dataset. B) Sample images of immunohistochemical staining and quantification of the SULT1E1^+^ subpopulation in grade I and grade II/III meningiomas. Scale bars: 20 µm. C) Sample images of immunohistochemical staining and quantification of the SULT1E1^+^ subpopulation in primary and recurrent meningiomas. Scale bars: 20 µm. D) Heatmap revealing significant outgoing signaling pathways of the MC SULT1E1^+^ subpopulation. E) The inferred ncWNT and NCAM signaling networks between different cell clusters. F) The inferred CSF signaling network between different cell clusters.

To validate our finding of the association between MC SULT1E1^+^ subpopulation and high‐grade meningiomas, we examined the signature‐gene expression of this subpopulation using the Gene Expression Omnibus (GEO) dataset. Many signature genes in this subpopulation, including SULT1E1, EDNRA, and EPHA7, were highly expressed in grade II/III meningioma samples (**Figure** **3**A, Figure S3A, Supporting Information). We also performed t‐SNE analysis after combining the data of our grade II meningioma sample (S2) and three high‐grade meningioma samples published by Wang et al. recently (MEN09, MEN13, and MEN108).^[^ ^11^ ^]^ These samples demonstrated similar distribution of meningioma cell clusters (Figure S3B, Supporting Information) and identical expression level of signature genes of MC SULT1E1^+^ subpopulation identified in our study (Figure S3C, Supporting Information). Notably, all tumor samples contained cells of MC SULT1E1^+^ subpopulation (Figure S3D, Supporting Information). Immunohistochemical staining of FFPE tissues showed a significantly increased number of MC SULT1E1^+^ cells in grade II/III and recurrent meningioma samples (Figure 3B,C) compared with grade I and primary meningioma samples. These results consolidated our finding of the unique SULT1E1^+^ subpopulation in high‐grade meningiomas.

We next performed cell‐cell interactome analysis with CellChat to better characterize the possible function of the MC SULT1E1^+^ subpopulation in interactions with other cell types, particularly macrophages. We uncovered five patterns for incoming and outgoing signaling (Figure S4A,B, Supporting Information). The MC SULT1E1^+^ subpopulation was very active in cell‐cell interaction, with many outgoing signals (Figure 3D). More specifically, MC SULT1E1^+^ exhibited strong cell‐cell communication with other MC subpopulations in many cancer‐associated signaling pathways, including the non‐canonical WNT and NCAM pathways (Figure 3E), suggesting that MC SULT1E1^+^ functions as a core modulator of tumor behavior by regulating other tumor cells. Another intriguing aspect of the cell‐cell interactome was the large number of outgoing signals in immune‐related signaling pathways (Figure 3D). CellChat predicted that MC SULT1E1^+^ acts as a source cell in the CSF signaling pathway, regulating the functions of macrophages, monocytes, and dendritic cells (Figure 3F). As key downstream effector cells, macrophages and monocytes also exhibited the properties of source cells in epidermal growth factor (EGF) and resisting signaling, interacting with meningioma or immune cells (Figure S4C,D, Supporting Information). The strong interactions between MC SULT1E1^+^ and macrophages and monocytes suggest that this unique subpopulation might be involved in M2‐like polarization and tumor development. These data demonstrate that the MC SULT1E1^+^ subpopulation is significantly associated with malignancy in meningiomas and may play a pivotal role in regulating the behavior of immune cells, notably M2‐like polarization of macrophages.

### 2.4 Meningioma Organoids Preserved the Intratumoral Heterogeneity of the Parental Tumors

**Figure 4:**
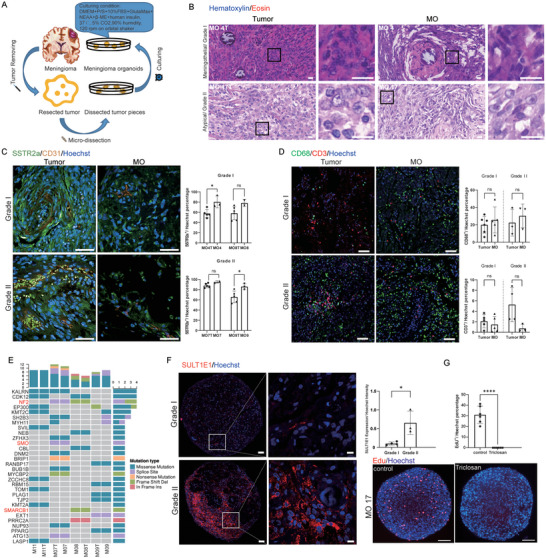
MOs maintain the histological features, molecular features, and intratumoral heterogeneity of the parent tumors. A) Schematic diagram of the process of culturing patient‐derived meningioma organoids. B) Representative hematoxylin and eosin (H&E)‐stained images of parental tumors and the corresponding MOs; scale bar: 20 µm. C) Representative images and quantification of immunostaining with anti‐SSTR2a and anti‐CD31 antibodies on grade I tumor/MO pairs (*n* = 4) and grade II tumor/MO pairs (*n* = 4); scale bar: 50 µm. D) Representative images and quantification of immunostaining with anti‐CD68 and anti‐CD3 antibodies on grade I tumor/MO pairs (*n* = 4) and grade II tumor/MO pairs (*n* = 4); scale bar: 50 µm. E) Heatmap indicating genetic variants of meningioma‐associated genes identified by whole‐exome sequencing of MOs and the corresponding parental tumors. F) Representative images and quantification of immunostaining with anti‐SULT1E1 antibody, revealing that grade II MOs retain the SULT1E1^+^ subpopulation; scale bars: 50 µm (left), 10 µm (right). G) Representative images and quantification of EdU staining of MOs after treatment with triclosan, showing decreased proliferation of MOs. Scale bars: 200 µm.

Tumor organoids have been established as a powerful tool for studying tumor heterogeneity.^[^ ^12^ ^]^ Therefore, we developed a novel patient‐derived meningioma organoid (MO) model to further characterize the MC SULT1E1^+^ subpopulation. After obtaining informed consent from the patients, fresh meningioma tumor samples were obtained during surgery and preserved. The bulk tumor was directly sheared into small pieces with a mean diameter of 1–2 mm with fine‐spring dissection scissors. Under customized culture conditions, the tumor pieces generally formed rounded MOs within 1 week (**Figure** **4**A). Because this strategy avoids mechanical and enzymatic dissociation of the tumor, most cell types and cell‐to‐cell interactions are well‐preserved. Moreover, the short preservation time and good quality of the tumor samples (no necrosis or bleeding) are conducive to the generation of MOs. The addition of fetal bovine serum (FBS) to the complete MO medium was essential for the formation of MOs; the tumor pieces did not form MOs in serum‐free medium containing B27 and N2. Using this protocol, we successfully developed 16 MOs (WHO grade I, 12; WHO grade II, 4) from 21 tumor samples (Table S1), with an overall success rate of 76.2%.

We first assessed the growth of MOs by monitoring the 2D area change, and MOs showed continuous growth during in vitro culture (Figure S5A, Supporting Information). Detection of incorporated EdU after 24 h of incubation showed that the proportion of proliferating cells (EdU^+^/Hoechst^+^ cells) in MOs remained relatively constant (Figure S5B, Supporting Information). We performed regular cell cryopreservation following the formation of MOs, and these MOs showed similar viability to continuously cultured MOs after recovery from liquid nitrogen storage (Figure S5C, Supporting Information), suggesting that MOs can be used to establish a live meningioma biobank.

We next investigated whether MOs could retain intratumoral heterogeneity of the corresponding parent tumor samples. The similarity of histological features between each MO and the corresponding tumor sample was confirmed by pathologists (Figure 4B and Figure S5D, Supporting Information). The MOs were cultured successfully and retained histopathological features identical to the diverse subtypes of the parent meningioma (meningothelial, fibrous, angiomatous, microcystic, and atypical). To further characterize cellular identities and the tumor microenvironment, we performed immunostaining analysis for several cell markers. Meningioma tumor cells (SSTR2a^+^) and vasculature (CD31^+^) were first identified in MOs (Figure 4C). Immune cells in parental tumors such as macrophages (CD68^+^) and T cells (CD3^+^) were also retained in the MOs (Figure 4D). To determine whether MOs maintained genomic alterations, we performed whole‐exome sequencing of four MOs and their corresponding parental tumors (Figure S5E, Supporting Information). The results showed that MOs could maintain most genetic variants found in the parental tumors, including mutations in NF2, SMO, and SMARCB1 (Figure 4E). Notably, MO models were successfully established from both NF2‐mutated and NF2‐wildtype tumor samples, indicating that this protocol is adaptable to meningiomas with different genetic landscapes.

One major advantage of this protocol is that it avoids potential clonal selection of regular 2D primary cell culture. Immunostaining confirmed that grade II MOs retained the MC SULT1E1^+^ subpopulation from the corresponding parental tumors (Figure 4F). Moreover, treatment with triclosan, a SULT1E1 inhibitor, caused a significant reduction in EdU^+^ cells in SULT1E1^+^ MOs but did not significantly reduce the expression level of SULT1E1 (Figure 4G and Figure S5F, Supporting Information). Due to the cellular heterogeneity, some MO individuals might be enriched with SULT1E1^+^ subpopulation cells. Meanwhile, some MOs didn't contain SULT1E1^+^ subpopulation cells and consisted with other tumor cell subpopulations. We performed scRNA‐Seq on those MO samples to investigate tumor cell heterogeneity (Figure S6A, Supporting Information). Sequencing results showed that multiple cell subtypes were retained in those MOs (Figure S6B, Supporting Information) and have similar signature gene expression with tumor cell subpopulations in the parental tumor (Figure S6C, Supporting Information). This result indicates that MO can maintain most of the tumor cell heterogeneity in the parental tumor.

In summary, we developed a novel protocol for the establishment of a patient‐derived MO model that retains the histological features, mutations, gene expressions, and most importantly, heterogeneous gene expressions, of the corresponding parent tumors.

### 2.5 SULT1E1^+^ MOs Displayed Brain Invasion Capabilities after Orthotopic Transplantation

Next, we sought to determine whether the SULT1E1^+^ subpopulation affected the behavior of MOs following orthotopic transplantation into immune‐deficient mice. We first performed subdural transplantation by removing parts of the meninges and brain tissue to create a cavity for the MOs (Figure S7A, Supporting Information). MOs derived from meningiomas with or without the MC SULT1E1^+^ subpopulation were transplanted. 2 months after transplantation, NMR T2‐weighted imaging (T2WI) was conducted for all mice, and most images showed transplanted MOs inside the skull (Figure S7B, Supporting Information). The overall success rate of subdural transplantation was 88.9%. We performed H&E staining of tissue dissected from these brains. Despite lesions to the meninges and brain tissue incurred during the transplantation procedure, SULT1E1^−^ MOs maintained a clear boundary with the brain tissue (Figure S7C, Supporting Information). However, the boundary between the SULT1E1^+^ MOs and brain tissue was disordered, indicating the possibility of brain invasion by the latter (Figure S7D, Supporting Information).

**Figure 5:**
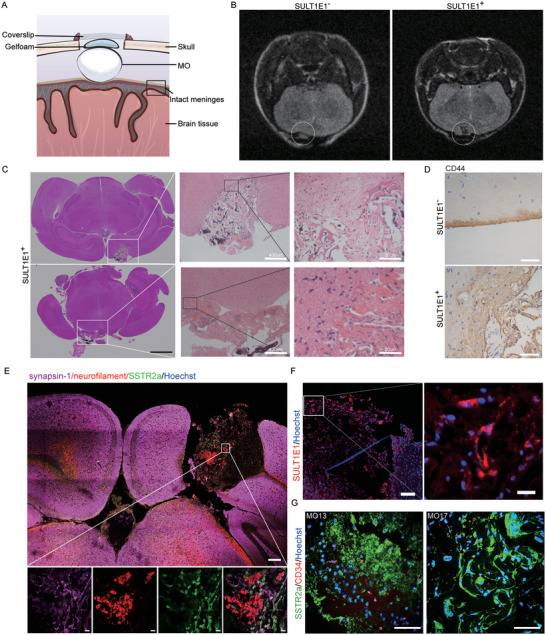
MOs containing the SULT1E1^+^ subpopulation retained the brain invasion capability of the parental tumors after epidural transplantation. A) Schematic diagram of epidural transplantation of MOs. B) Representative T2‐weighted NMR images (T2WI) of mouse heads 2 months after epidural transplantation. C) Representative H&E staining of orthotopic tumor models using epidural‐transplanted MOs. D) Representative immunohistochemistry images of CD44 in orthotopic tumor models. Disruption of the normal tumor–brain boundaries is shown as a discontinuous line; scale bar: 50 µm. E) Representative images if immunostaining with anti‐synapsin‐1, anti‐neurofilament, and anti‐SSTR2a antibodies of SULT1E1^+^ MO orthotopic tumor models. Scale bar: 200 µm (top), 10 µm(bottom). F) Representative confocal images of immunostaining with anti‐SULT1E1 antibody, confirming that transplanted MOs retain the SULT1E1^+^ subpopulation; scale bars: 100 µm (left), 20 µm (right). G) Representative confocal images of immunostaining with anti‐SSTR2a and anti‐CD34 antibodies; scale bar: 50 µm.

To further explore the biological behavior of MOs in vivo, we conducted epidural transplantation of SULT1E1^+^ and SULT1E1^−^ MOs. Here, MOs were transplanted into the space between the skull and meninges of immune‐deficient mice without damaging the meninges or brain tissue (**Figure** **5**A). The same NMR scanning procedure was performed 2 months after transplantation. The results showed clear outlines between the MOs and the brains of the mice transplanted with SULT1E1^−^ MOs, but abnormal brain morphology in the mice transplanted with SULT1E1^+^ MOs (Figure 5B). Generally, the success rate of epidural transplantation was significantly lower than that of subdural transplantation (41.2% versus 88.9%, Table 2). Further dissection showed that the SULT1E1^+^ MOs tightly adhered to the brain tissue, whereas the SULT1E1^−^ MOs remained isolated from the meninges (Figure S7E, Supporting Information). H&E staining of xenografts transplanted with SULT1E1^+^ MOs was observed in the subarachnoid space and exhibited abnormal brain‐organoid boundaries (Figure 5C). Brain invasion by SULT1E1^+^ MOs was confirmed by immunohistochemistry using anti‐CD44 antibody (Figure 5D). Immunostaining with anti‐synapsin‐1, anti‐neurofilament, and anti‐SSTR2a antibodies clearly showed destruction of brain cortex and residual cortex tissue surrounded by meningioma tumor cells, further proving the brain invasion of SULT1E1^+^ MOs (Figure 5E). Immunostaining also showed that the MC SULT1E1^+^ subpopulation was retained in all brain‐invasive MOs (Figure 5F). Additionally, host‐origin vasculature was found in most transplanted MOs, indicating neo‐angiogenesis after orthotopic transplantation (Figure 5G).

Therefore, we introduced two new protocols for establishing a patient‐derived orthotopic tumor model for meningiomas using MOs. Following transplantation, the MOs containing the MC SULT1E1^+^ subpopulation had the capacity for brain invasion, a unique biological behavior identical to that of high‐grade meningiomas.

### 2.6 High‐Throughput Screening for Therapeutic Targets in the MC SULT1E1^+^ Subpopulation Using the MO Model

**Figure 6:**
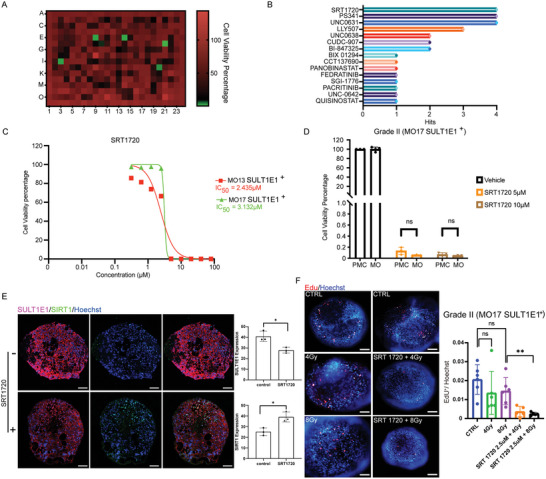
SRT1720 shows potent inhibition targeting the MC SULT1E1^+^ subpopulation in MOs. A) Representative heatmap showing the positive hits of high‐throughput drug screening. Green color indicates the location of the positive hit with cell viability below 10%. B) The top 13 compounds of 305 epigenetic inhibitors ranked by hit numbers. C) Cell viability curve for SRT1720 for two SULT1E1^+^ meningioma primary cell lines. D) Cell viability plots for specific concentrations of SRT1720 for primary meningioma cell (PMC) lines and the corresponding MOs. E) Representative confocal images and quantification of slices immunostained with anti‐SULT1E1 and anti‐SIRT1 antibodies after incubation with 2.5 µm SRT1720 for 72 h; scale bar: 100 µm. F) EdU staining of MOs showing a significant reduction in cell proliferation after a combination of SRT1720 treatment and radiotherapy.

Because our data suggested that the MC SULT1E1^+^ subpopulation might play a key role in the malignancy of high‐grade meningiomas, we next investigated potential therapeutic strategies by inhibiting this unique subpopulation. To this end, a panel of 305 epigenetic compounds was tested at a 10 µm concentration on four primary MC lines derived from SULT1E1^+^ high‐grade meningioma samples. A positive hit was defined as a cell survival rate <10% of the DMSO control (**Figure** **6**A). The top three compounds ranked by total hit number were SRT1720, PS341, and UNC0631 (Figure 6B).

SRT1720 is a specific synthetic activator of SIRT1 (silent mating type information regulation 2 homolog 1; Sirtuin 1),^[^ ^13^ ^]^ PS341 (bortezomib) is a selective proteasome inhibitor that targets the 26S proteasome,^[^ ^14^ ^]^ and UNC0631 is a potent inhibitor of histone methyltransferase G9a.^[^ ^15^ ^]^ The inhibitory effect of these three compounds was further confirmed in three MO‐derived meningioma primary cell lines (Figure 6C, Figure S8A,B, Supporting Information). We next treated similar‐sized SULT1E1^+^ MOs with these three compounds at concentrations that had shown significant inhibition of the primary meningioma cell lines (PMCs). Surprisingly, the MOs exhibited obvious resistance to PS341 and UNC0631 (Figure S8C, Supporting Information), and the response of MOs derived from the same patient was also variable, suggesting that MOs represent chemoresistance and heterogeneity of the original meningioma tumor. However, SRT1720 showed an inhibitory effect on SULT1E1^+^ MOs similar to that on 2D cell lines (Figure 6D). Immunostaining confirmed that the expression of SULT1E1 was significantly downregulated in SULT1E1^+^ MOs following SRT1720 treatment (Figure 6E). Downregulation of SULT1E1 was further validated by western blotting (Figure S6D, Supporting Information). Additionally, combination therapy with 2.5 µm of SRT1720 and radiation significantly inhibited cell proliferation in SULT1E1^+^ MOs (Figure 6F). These data revealed that SRT1720 has potential value as a therapeutic agent and radiation sensitizer because of its ability to effectively inhibit the MC SULT1E1^+^ subpopulation in high‐grade meningiomas.

## 3 Discussion

In the most recent 2021 WHO classification of tumors of the central nervous system, meningiomas are still graded based on their mitotic rate, brain invasiveness, and histological features.^[^ ^16^ ^]^ A few molecular biomarkers have been associated with the grading and subtyping of meningiomas, such as SMARC1 (clear cell subtype),^[^ ^17^ ^]^ KLF4/TRAF7 (secretory subtype),^[^ ^18^ ^]^ and the homozygous deletion of CDKN2A/B (anaplastic).^[^ ^6a^ ^]^ DNA methylation profiling has also been used to predict the recurrence of meningiomas.^[^ ^19^ ^]^ However, these findings are inadequate to unravel the mechanism underlying the malignancy of high‐grade meningiomas and provide potential targets for systemic treatment. In the current study, scRNA‐Seq was employed to uncover the intratumoral heterogeneity of meningiomas. We examined the cellular difference between benign and high‐grade meningiomas and identified a unique subpopulation of tumor cells that may drive the malignancy and aggressiveness of high‐grade meningiomas. This finding may deepen our understanding of the mechanisms of tumor initiation, development, and invasion in high‐grade meningioma.

The tumor immune microenvironment has gained intense attention owing to its crucial contribution to tumor progression.^[^ ^20^ ^]^ In two recent studies, single‐cell RNA‐seq was first applied to meningioma samples, revealing multiple tumor‐infiltrating immune cell subpopulations in meningiomas.^[^ ^11^, ^21^ ^]^ However, the differences in cell composition between benign and high‐grade meningiomas has not been investigated yet. Here we further validated the increased macrophage proportion and M2‐like polarization status in high‐grade meningiomas from a single‐cell perspective, which is in accordance with previous reports showing that M2‐like macrophages promote the development and invasiveness of meningiomas.^[^ ^10^, ^22^ ^]^ The predominance of M2‐like macrophages suggests an immune‐suppressive microenvironment in high‐grade meningiomas,^[^ ^23^ ^]^ offering potential targets for immune therapy. Several preliminary studies and clinical trials have been conducted to evaluate the potency of immunotherapy for high‐grade or recurrent meningiomas and have shown promising results.^[^ ^24^ ^]^

Recently, tumor heterogeneity has been recognized as a major factor in resistance to chemotherapy. radiation therapy and tumor recurrence.^[^ ^7^, ^25^ ^]^ Among our key findings was the unique association between the MC SULT1E1^+^ subpopulation and high‐grade meningiomas. We validated the clinical significance of the MC SULT1E1^+^ subpopulation in high‐grade meningiomas by using both GEO datasets and immunostaining of FFPE tissues. Moreover, the results of the cell‐cell communication analysis implied that this subpopulation drives malignancy by modulating the functions of other tumor and immune cells. Both ncWNT and NCAM signaling pathways, in which the MC SULT1E1^+^ subpopulation shows strong interactions with other tumor cells, plays important roles in the morphogenesis, development, and invasiveness of tumors.^[^ ^26^ ^]^ We observed a significantly higher proportion of this subpopulation in the recurrent meningioma samples. Combining these results, we speculated that the MC SULT1E1^+^ subpopulation directly mediates oncogenesis and development of high‐grade meningiomas. We noticed that a minority of grade I meningioma samples also contained a few SULT1E1^+^ cells. Recently, many studies have reported that a group of patients with grade I meningiomas have significantly worse outcomes than others.^[^ ^27^ ^]^ Considering the association between the SULT1E1^+^ subpopulation and tumor recurrence, it will be important to determine the difference in prognosis between grade I meningiomas with and without the SULT1E1^+^ subpopulation in future research. Recently, integrative molecular classifications based on DNA methylation profiling for meningiomas have been developed rapidly, which have all been proven to better predict clinical outcomes.^[^ ^21^, ^27^, ^28^ ^]^ Positivity of SULT1E1^+^ subpopulation might be a new factor to consider for the risk evaluation of the patients with meningioma.

In addition to other tumor cell subpopulations, the SULT1E1^+^ subpopulation may also modulate the activity of immune cells in the tumor. In our study, cell‐cell communication analysis predicted a strong interaction between the SULT1E1^+^ subpopulation and macrophages via the CSF1‐CSF1R signaling network. CSF1‐CSF1R signaling is known to directly trigger M2‐like polarization of macrophages, and consequently reshape the tumor environment.^[^ ^29^ ^]^ This is in accordance with our finding of the dominant M2‐like polarization status of macrophages in high‐grade meningiomas. These results suggested that the SULT1E1^+^ subpopulation in high‐grade meningiomas may directly contribute to the formation of an immunosuppressive environment, which is a common characteristic of aggressive tumors.^[^ ^30^ ^]^ Targeting the CSF1‐CSF1R axis has been suggested as a potential treatment strategy for malignant meningiomas,^[^ ^24b^ ^]^ and combination therapies targeting both CSF1‐CSF1R axis and SULT1E1^+^ subpopulation may be a more effective immunotherapy for high‐grade meningiomas.

To better study the characteristics of the SULT1E1^+^ subpopulation, we developed a novel protocol for establishing a patient‐derived MO model based on that outlined by Jacob et al.^[^ ^31^ ^]^ Compared to the protocols for other MO models reported previously,^[^ ^32^ ^]^ one major advantage of our protocol is that it avoids the enzymatic digestion of tumor samples into single cells, thereby ensuring the preservation of most cell types in the MO model. As shown by immunostaining, the MOs maintained a complex cell composition, including tumor cells, vascular endothelial cells, tumor‐infiltrating macrophages, and T lymphocytes, all of which were similar to those of the parental tumor.^[^ ^33^ ^]^ This suggests that MOs could be a powerful tool for research into the tumor microenvironment of meningiomas. Most importantly, the MOs retained the SULT1E1^+^ subpopulation from the corresponding parental tumor. SULT1E1^+^ MOs showed the ability to invade the meninges and brain tissue after orthotopic transplantation. Brain invasion is a key feature of high‐grade meningiomas and a strong predictor of tumor recurrence.^[^ ^34^ ^]^ Currently, only a limited number of experimental systems are available to study brain invasion by meningiomas.^[^ ^35^ ^]^ Our findings may provide additional insights into the factors driving brain invasion and a reliable in vivo model to support future studies.

The development of traditional molecular meningioma therapies targeting genetic variants has not produced satisfactory results.^[^ ^5^, ^24^, ^36^ ^]^ Triclosan significantly inhibited the proliferation of tumor cells in SULT1E1^+^ MOs, however its clinical application for the treatment of high‐grade meningioma is limited by the potential oncogenic risks.^[^ ^37^ ^]^ Here, we present another potential therapeutic strategy that effectively inhibits the SULT1E1^+^ subpopulation. Our results suggest that SRT1720 could be used as a systemic treatment or a radiotherapy sensitizer to suppress tumor growth. SRT1720 inhibits bladder cancer growth in organoids and murine models through the SIRT1/HIF axis.^[^ ^38^ ^]^ The detailed mechanisms underlying the inhibitory and radio‐sensitization effects of SRT1720 on the SULT1E1^+^ subpopulation remain unclear and warrant further research.

## 4 Conclusion

In summary, we used scRNA‐Seq technology to identify a unique subpopulation of tumor cells mostly found in grade II and III meningiomas. A comprehensive analysis suggests that this subpopulation, SULT1E1^+^, plays an important role in the genesis and recurrence of high‐grade meningiomas. Moreover, we established a novel patient‐derived MO model that mimicked the intratumoral heterogeneity and biological behavior of meningiomas. By targeting the SULT1E1^+^ subpopulation, we identified SRT1720, an SIRT1 agonist, as a potential systemic treatment and radiation sensitizer for high‐grade meningiomas.

## 5 Experimental Section

### Human Tumor Specimens

All meningioma samples were collected from the Department of Neurosurgery, Xiangya Hospital after informed consent from the patients. Tumor samples without obvious hemorrhage, necrosis, or electrical burn were collected during the surgery and placed immediately in MACS Tissue Storage Solution (Miltenyi) for single‐cell isolation or DMEM medium with 1X PenStrep (Thermo Fisher Scientific) for organoid culture and kept at 4 °C until further processing. For a higher rate of success, it was crucial to reduce the time of storing the tumor samples at 4 °C.

### Single‐Cell Isolation

The tumor samples were washed with cold DPBS three times and then transferred to a sterile glass dish with cold DMEM medium and 1X PenStrep in a biosafety cabinet. The tumor sample was dissected into small pieces first with an average diameter of 0.5 cm by dissection scissors and further dissected into smaller ones with a diameter of 1 mm with fine‐spring dissection scissors and micro forceps (RWD Life Science CO.). The pieces were transferred to the gentle MACS C Tubes(Cat# 130‐096‐334, Miltenyi Biotec), with 5 mL digestive enzyme from Tumor Dissociation Kit (Cat# 130‐095‐929,MiltenyiBiotec). Then the tissues were digested into single‐cell suspension using the gentleMACS Dissociator (Cat# 130‐093‐235, MiltenyiBiotec). Viability was confirmed to be >85% in all samples using trypan blue (ThermoFisher Scientific). Cell suspensions were filtered using a 70 mm filter (ThermoFisher Scientific), and single‐cell suspension were pelleted and re‐suspended in PBS with 1% BSA (Sigma‐Aldrich, St. Louis, MO).

### Single Cell Sequencing

scRNA‐seq was performed with the 10X Chromium 30 v2 kit (10X Genomics, Pleasanton, CA) following the manufacturer's protocol. Number of captured cells ranged from 2000 to 10 000. Sequencing libraries were prepared following manufacturer's protocol. Sequencing was performed on a NovaSeq 6000 (Illumina, Inc., San Diego, CA). Raw sequencing data was processed with the cellranger pipeline (version 2.1.0, 10X Genomics) and mapped to the hg19 reference genome to generate matrices of gene counts by cell barcodes.

### Whole Exome and Targeted Sequencing

Genomic DNA of tissue samples were extracted using the QIAamp AllPrep DNA/RNA Mini Kit (Cat# 80 204, QIAGEN) according to the manufacturer's specifications including the steps of DNA adsorption, purification, and collection in columns. The quality of isolated genomic DNA was verified by using these two methods in combination: 1) DNA degradation and contamination were monitored on 1% agarose gels. DNA concentration was measured by Qubit DNA Assay Kit in Qubit 2.0 Flurometer (Life Technologies, CA, USA). A total amount of 1 µg DNA per sample was used as input material for the DNA library preparations. Sequencing library was generated using CLEANNGS DNA kit following manufacturer's recommendations and index codes were added to each sample. Briefly, genomic DNA sample was taken and enzymatically disrupted to a size of 180–280 bp. Then DNA fragments were endpolished, A‐tailed, and ligated with the full‐length adapter for Illumina sequencing, followed by further PCR amplification. After PCR reaction, libraries were hybridized with liquid phase with biotin labeled probe(SureSelectXT Human All Exon V6), then magnetic beads were used with streptomycin to capture the exons of genes. Captured libraries were enriched in a PCR reaction to add index tags to prepare for sequencing. Products were purified using AMPure XP system (Beckman Coulter, Beverly, USA) and quantified using the Agilent high sensitivity DNA assay on the Agilent Bioanalyzer 2100 system. The clustering of the index‐coded samples was performed on a cBot Cluster Generation System using Novaseq5000/6000 S4 Reagent Kit (Illumina) according to the manufacturer's instructions. After cluster generation, the DNA libraries were sequenced on Illumina NovaSeq 6000 platform and 150 bp paired‐end reads were generated. Clean reads were compared with reference human genome (UCSC hg19) by using BWA software, and the results were converted into bam format and sorted by samtools software. Finally, basic information statistics and map comparison statistics were conducted. Mutect2 software was used to do variant calling and identify Somatic SNV/InDel, Enliven was performed to do annotation for SNV/InDel. Big data of WES sequencing data of healthy people obtained from BerryGenomics Company was applied as general background to identify potential pathogenic mutations.

### Single‐Cell Data Processing

Gene‐barcode counts matrices were analyzed with the Seurat R package (version 4.0.5). Cells with <500 or >4000 genes detected and >20% mitochondrial gene mapped reads were filtered from downstream analyses. All samples were normalized and identified variable features independently with NormalizeData function and FindVariableFeatures function. Then identify anchors using the FindIntegrationAnchors function, which takes a list of Seurat objects as input, and use these anchors to integrate the datasets together with IntegrateData function. Dimensionality reduction was then performed using PCA and UMAP plots were generated by the RunUMAP function with the first 20 PCs as input, determined by visualizing the drop off in PC variance explained using the ElbowPlot function in Seurat.

### Cell Type Annotation

In order to determine the cell types in meningioma samples, this work combined unsupervised clustering and differential expression to compare the most differentially expressed genes with cell type specific marker reported in literature. Through this the constituent cell types and further analysis cellular subtypes were identified by isolating meningioma cells subsets and re‐analyzing with the same approach. For cell type annotation shown in Figure 1B, low‐resolution clustering was performed by FindClusters function with the first 20 PCs and resolution 0.3 to generate 14 clusters. Differential expression was performed using the FindAllMarkers function in Seurat with default parameters. Two of these clusters (clusters 7, 13) highly expressed epithelial‐associated genes such as CLU, PTGDS, FN1, and COL1A2 and were therefore merged and inferred to be meningioma cells. Macrophages made up of four clusters (clusters 0, 5. 6, 10) and highly expressed CD163 and MRC1. Other clusters highly expressed markers specific for T cells (CD3D, CD8A); NK cells (GNLY, NKG7, KLRD1), B cells (CD79A, IGLL5, MS4A1), dendritic cells (HLA‐DPA1, CD74, RGS1), monocytes (LYZ, FCN1, VCAN), and neutrophils (FCGR3B, IFITM2, CD55).

For the further clustering of meningioma cells, the top 15 PCs were selected with resolution 0.4. To define feature genes in meningioma cells, differential expression analysis between meningioma cells was carried out using FindAllMarkers function, with log‐scaled fold change ≥0.25 and *p* value <0.05 (Wilcoxon Rank Sum test).

### Single Cell CNV Calling

To confirm the identified meningioma cells were malignant cells with chromosomal copy number variations, the inferCNV R package was used to infer the genetic profiles of each cell based on the average expression of large genes sets (101 genes) in each chromosomal region of the tumor genome compared to normal cells.

### Meningioma Cell Pseudotime Analysis

The pseudotime trajectory of meningioma cells was inferred by using Monocle. The FindAllMarkers function implemented in the Seurat package was used to identify the top 50 variable feature genes with a *q*‐value <0.01 from each meningioma cells’ cluster and genes were used to order the cells in pseudotime trajectory analysis. After the pseudotime trajectories were constructed, differentially expressed genes along the pseudotime trajectory were analyzed using the differentialGeneTest function implemented in the monocle package.

### Definition of Cell Scores and Signature

To compare the M1/M2 polarization of myeloid cells (macrophages/monocytes) in Grade I and Grade II meningiomas, AddModuleScore function implemented in the Seurat package was applied to calculate the average expression levels of M1/M2 genes. The M1/M2 gene sets were described by Azizi et al. (2018) (Azizi, E., Carr, A.J., Plitas, G., Cornish, A.E., Konopacki, C., Prabhakaran, S., Nainys, J., Wu, K., Kiseliovas, V., Setty, M., et al. (2018). Single‐Cell Map of Diverse Immune Phenotypes in the Breast Tumor Microenvironment. Cell 174, 1293–1308).

### Gene Ontology Analysis

To analyze and visualize functional profiles of feature genes (GO biology process) from meningioma samples’ clusters, meningioma cells’ clusters, and meningioma cell pseudotime trajectory, the clusterProfiler package was used. Each cluster's feature genes were derived by FindAllMarkers function implemented in the Seurat package which were the top 50 variable feature genes with a *q*‐value <0.01. Differentially expressed genes along the pseudotime trajectory were derived as described before.

### Cell‐Cell Communication Analysis

CellChat package was used to identify the potential interactions between Grade II meningioma clusters. The gene expression matrix of Grade II meningioma was inputted and analyzed based on the known ligand–receptor interaction information encapsulated in CellChat package, which computed the probability and significance of communications.

### Transcriptomics Data Collection and Processing

Human meningioma tissue transcriptomics data (GSE136661) were found in the publicly available repository Gene Expression Omnibus (GEO), which contained WHO grade information. Raw data and clinical information were downloaded using getGEO function implemented in GEOquery packages. Further analysis was conducted using DESeq2 package following standard procedure.

### Generation of MOs

The tumor samples were washed with cold DPBS three times and then transferred to a sterile glass dish with cold DMEM medium and 1X PenStrep in a biosafety cabinet. The tumor bulk was dissected into small pieces first with an average diameter of 0.5 cm by dissection scissors and further dissected into smaller ones with a diameter of 1 mm with fine‐spring dissection scissors and micro forceps (RWD Life Science CO.). Horizontally pulling or repeated cutting should be avoided to maintain the integrity of tumor pieces. Tumor pieces were washed three times with room temperature DPBS to remove the debris and incubated in 1X RBS lysis buffer (Thermo Fisher Scientific) for 15 min at room temperature. RBS lysis buffer was aspirated and tumor pieces were washed three times with room temperature DMEM medium with 1× PenStrep. MO medium stock was prepared by adding 50 mL fetal bovine serum, 5 mL 100X PenStrep, 5 mL 100X GlutaMax (Thermo Fisher Scientific), 5 mL 100X non‐essential amino acid solution (NEAA), and 125 µL human insulin solution (Sigma Aldrich) into 450 mL DMEM medium. Complete MO medium was made by aliquoting 50 mL of MO medium stock into conical tubes and adding 50 µL of 2‐Mercaptoethanol. MO medium stock and complete MO culture medium can be stored at 4 °C for up to a month and a week, respectively. Tumor pieces were resuspended with complete MO medium, 20–30 pieces were placed in a cryogenic storage vial and snap frozen in liquid nitrogen for further sequencing. Another 20–30 pieces were transferred into 100 mm TC treated cell culture dishes with 10 mL DMEM/10%FBS medium for the culture of primary 2D cell lines. The rest of the tumor pieces were distributed into ultra‐low attachment 6‐well culture plates (Corning) with 4 mL of complete MO medium for each well and cultured on an orbital shaker rotating at 120 rpm within a 37 °C, 5% CO2, and 90% humidity sterile incubator. The density of 20–30 pieces for each well is appropriate for the formation of MOs. Overcrowdedness will lead to the adhesion of tumor pieces and the irregular shape of MOs. The medium was changed every 3 days by aspirating 3 mL of the medium for each well and then adding 3 mL of fresh complete MO medium. Generally, the tumor pieces formed organoids with rounded shapes within 1 week.

### Biobanking and Recovery for MOs

Within 1 month of culture, the medium was aspirated and MOs were resuspended with complete MO medium containing 10% of DMSO and placed in cryovials with 20–30 MOs and 1.5 mL of medium per vials. After 15 min of incubation at room temperature, cryovials were transferred into BeyoCool Cell Freezing Container and placed in a −80 °C freezer. Frozen MOs were transferred into a liquid nitrogen tank for long‐term storage. For recovery, frozen cryovials were thawed in a 37 °C water bath, and MOs were quickly transferred into a 15 mL conical tube. The medium was aspirated and 10 mL of fresh complete MO medium was added into the tube. After washing three times with fresh complete MO medium, MOs were plated in ultra‐low attachment 6‐well culture plates and cultured as described above. The cell viability of recovered MOs was tested with Calcein/PI Cell Viability/Cytotoxicity Assay Kit (Beyotime) according to manufacturer's instruction.

### Tissue Processing and HE Staining

Formalin‐fixed paraffin‐embedded (FFPE) tissue sections mounted on microscope slides of corresponding tumor samples were acquired from the pathological department, Xiangya hospital. The MOs were incubated with 10 µm EdU(Beyotime) in the complete MO medium for 24 h before fixation. EdU‐labeled MOs or mouse brains of PDX models after dissection were fixed in 4% paraformaldehyde(PFA) for 30 min or overnight, respectively, and then dehydrated and embedded with paraffin. FFPE tissues were sectioned at a thickness of 10 µm and deparaffinized accordingly. Standard H&E staining was performed for both slides of MOs and corresponding tumor tissues. Representative H&E staining images were taken with Olympus BX63 microscope.

### EdU Incubation and Staining

Incorporation and detection of EdU were performed with BeyoClick EdU Cell Proliferation kit with Alexa Fluor 647 (Beyotime). Similarly sized MOs were incubated with 10 µm EdU in an ultra‐low attachment 24‐well culture plate. 24 h later, the culture medium was aspirated and 500 µL of 4% PFA was added into the well for 30 min at room temperature. After washing with DPBS, MOs were permeabilized with PBS containing 0.3% Triton X‐100 for 30 min. After washing with DPBS, the click reaction solution was prepared according to the manufacture's manual, 500 µL of click reaction buffer was added into each well, the plate was covered with aluminum foil and placed at an orbital shaker. Incubation was performed for 30 min at room temperature. After washing with PBS containing 3% bovine serum albumin(BSA) for three times, cell nuclei were labeled by adding 500 µL of PBS containing 1X Hochest 33 342 into each well and incubating for 15 min at room temperature. Extra Hochest 33 342 was removed by washing with DPBS. One MO and 100 µL of DPBS were transferred to one well of 96‐well black flat bottom plates(Corning) for imaging. Images were taken with Opera Phenix High Content Screening System (Perkin Elmer), the distance of adjacent layer was set as 10 µm, 10 layers were imaged for one MO and then merged into one image. Unpaired *t*‐test was applied to compare the difference between groups.

### Immunofluorescence Staining

For FFPE tissue sections, heat mediated antigen retrieval was performed with citrate pH 6 in a microwave oven for 30 min after deparaffinization. After washing with PBS, tissue sections were permeabilized and blocked using a solution with TBS containing 0.3% Triton X‐100 and 5% donkey serum (D‐TBST solution) for 1 h at room temperature. Then the tissue sections were incubated with primary antibody() diluted with D‐TBST solution at 4 °C overnight. After washing with TBS containing 0.05% Tween‐20 for three times, the tissue sections were incubated with secondary antibody and Hochest (1:1000) diluted with D‐TBST solution at room temperature for 1.5 h. After washing with TBST, slides were mounted in mounting solution, coverslipped, and sealed with nail polish. Staining of EdU incorporation was performed before the blocking with BeyoClick EdU Cell Proliferation kit with Alexa Fluor 647 according to the manufacture's manual. Confocal images were taken with Opera Phenix High Content Screening System (Perkin Elmer). The scanning and analysis of IHC staining was performed by Wuhan Servicebio technology company with Aipathwell software. After intensity and area were calculated, the H‐Score was calculated with the formula below:

(1) $$\begin{array}{ccc} H-\mathrm{SCORE}=\sum \left(\mathrm{pi}\times i\right) & = & \left(\mathrm{percentage}\;\mathrm{of}\;\mathrm{weak}\;\mathrm{intensity}\times 1\right)\\ & & +\left(\mathrm{percentageof}\mathrm{moderate}\mathrm{intensity}\times 2\right)\\ & & +\left(\mathrm{percentage}\mathrm{of}\mathrm{strong}\mathrm{intensity}\times 3\right) \end{array}$$

### MOs Growth Analysis

TO measure the changes of 2D area of MOs, similarly‐sized MOs with rounded shapes were placed into ultra‐low attachment 96‐well culture plates. For each well, one MO and 100 µL of complete MO medium were added with an end‐cut P200 pipette tip. A bright‐field image of every single MO was taken with Opera Phenix High Content Screening System (Perkin Elmer). The 2D area of each MO was calculated in the ImageJ with the wand tool. The 2D area for individual MO at each time point was divided by the 2D area at time 0 to calculate the change rate.

### Animals and Orthotopic Transplantation of GBOs

Female immunodeficient M‐NSG mice (3–4 weeks old) were obtained from the Shanghai Model Organisms. All animal experiments were carried out according to the guidance of the ethics committee of Sun Yat‐sen University. NSG mice were induced into anesthesia and maintained with inhaled isoflurane. The animal head was fixed in a stereotactic frame and the body temperature was maintained at 37°C using an electric heating pad. A small incision was made with a surgical scalpel at the skin above the skull. A 3 mm diameter square skull window was made by carefully drilling, the drilling site is before the lambda and lateral to the sagittal suture. For epidural transplantation, a 1–2 mm diameter MO was picked by micro forceps and placed at the surface of meninges. A small piece of wet gelform was placed on the MO and sealed by a 3 mm sterilized round coverslip and Vetbond Tissue Adhesive (3 m). Dental cement was applied onto the surrounding skull to further immobilize the coverslip. For subdural transplantation, the lesion was made with capsulorhexis forceps and p200 tip connected to the vacuum machine to remove the meninges and cortex tissue. After controlling the bleeding with a small piece of gelfoam, a 1–2 mm diameter MO was picked by micro forceps and placed in the hole. The sealing procedure was the same as described above. The skin wound was closed with Vetbone Tissue Adhesive. Animals were grouped‐housed with up to five mice per cage. 2 months after the transplantation, mice were scanned with MRI and sacrificed. Brains were carefully removed from the skull. Bright‐field images of brains and skulls were taken with stereoscopic microscope. Brains were fixed in 4% PFA at 4 °C overnight and then dehydrated and embedded with paraffin for further sectioning.

### High Throughput Drug Screening and Dose‐Response Analysis

For high‐throughput screening, a panel of 305 epigenetic compounds was used to screen meningioma cells for drug sensitivity. Meningioma cells were plated in 384 well black microplates with the density of 0.5k cells and 30 µL of medium for each well and allowed for adhesion in the incubator overnight. 305 compounds were added into the wells to achieve the final concentration of 10 µm. Cells were incubated for 72 h and then cell viability was detected with CellTiter‐Glo Assay(Promega). The compound was defined as a positive hit if the cell viability was below 10% comparing to the DMSO control. For the dose‐response experiment, meningioma cells were plated in 96 well plates with the density of 3k and 100 µL of the medium in triplicate and allowed for adhesion in the incubator overnight. 100 µL of medium containing 2× concentration of PS‐341, SRT‐1720, and UNC‐0631 was added into the corresponding well. Cells were incubated for 72 h and then the cell viability was detected with CellTiter‐Glo Assay. Dose‐response curves were generated by a nonlinear regression analysis using Graphpad Prism, version 9. For the treatment of specific compounds on MOs, each compound was diluted into final concentration with complete MO medium and distributed into ultra‐low attachment 96‐well culture plates. Similar‐sized MOs were distributed into each well and incubated for 72 h and then corresponding assays of detection were performed.

### Radiation and Combined Treatments of MOs

For radiation experiments, MOs were irradiated at a total dose of 4 or 8 Gy. After the radiation, MOs were incubated with 10 µm of EdU for 24 h. Detection of EdU‐labeled cells is introduced above. For the combined treatments, MOs were incubated with 2.5 µm of SRT‐1270 for 72 h before the radiation. Unpaired *t*‐test was applied to compare the difference between groups.

### Ethics Approval Statement

All animal experiments were approved by the Institutional Animal Care and Use Committee of Sun Yat‐Sen University and carefully conducted according to the Guide for the Care and Use of Laboratory Animals.

## Conflict of Interest

The authors declare no conflict of interest.

## Author Contributions

M.H. and S.X. contributed equally to this work and share the first authorship. M.H. and S.X. performed the experiments and conducted bioinformatics analysis of the sequence data. Y.L., C.Q., L.S., J.S., Z.J., and Y.H. collected tumor samples and clinical data. X.Z. and L.Q. managed the mouse models and performed MRI scanning. W.L., Q.L., and W.Z. designed the experiments, interpreted the data, wrote the manuscript, and supervised the study.

## Supporting information

Supporting Information

## Data Availability

The data that support the findings of this study are available from the corresponding author upon reasonable request.
