# Supplementary material for: Novel Human Meningioma Organoids Recapitulate the Aggressiveness of the Initiating Cell Subpopulations Identified by ScRNA‐Seq
Source: Adv Sci (Weinh). 2023 Mar 30;10(15):2205525. doi: 10.1002/advs.202205525 (PMC10214266; doi:10.1002/advs.202205525)
Supplement: Supplementary file 1 — Supporting Information [file ADVS-10-2205525-s001.pdf]

Supporting Information

Novel Human Meningioma Organoids Recapitulate the Aggressiveness of the Initiating Cell Subpopulations Identified by ScRNA-Seq

Meng Huang, Shao Xu, Yuzhe Li, Li Shang, Xiudan Zhan, Chaoyin Qin, Jun Su, Zijin Zhao, Yi He, Lina Qin, Wei Zhao*, Wenyong Long*, and Qing Liu*

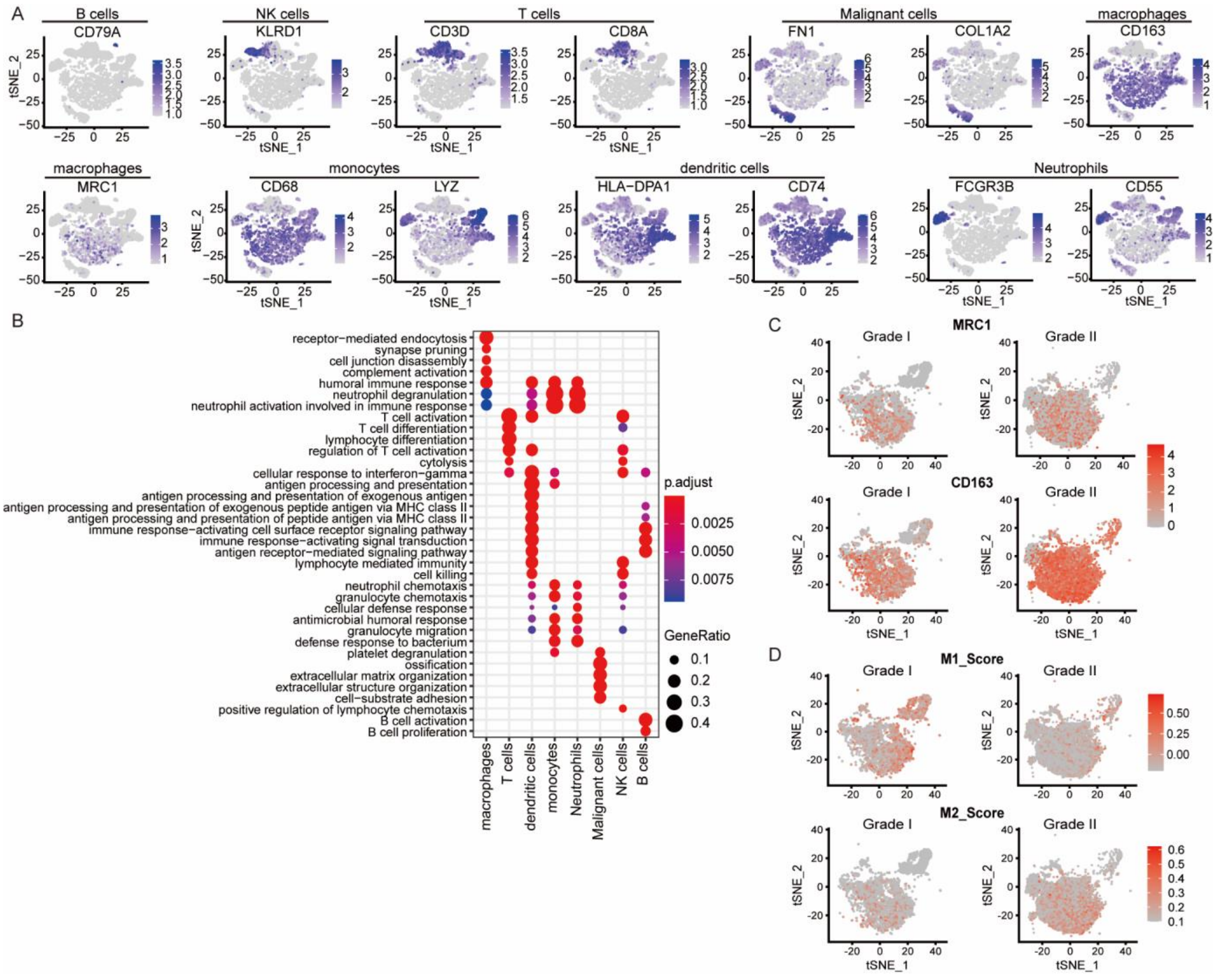

Figure S1. Signature gene expression of different cell clusters across meningioma tumor samples

A. *t*-distributed stochastic neighbor embedding (*t*-SNE) plots showing the expression of established cell markers in cell clusters. B. Dot plot showing the gene ontology term enrichment of the top 50 signature genes in different cell clusters. C. t-SNE plots of macrophage and monocyte clusters, colored by expression level of M2-polarization marker MRC1 and CD163. D. t-SNE plots of macrophage and monocyte clusters, colored by M1 and M2 scores.

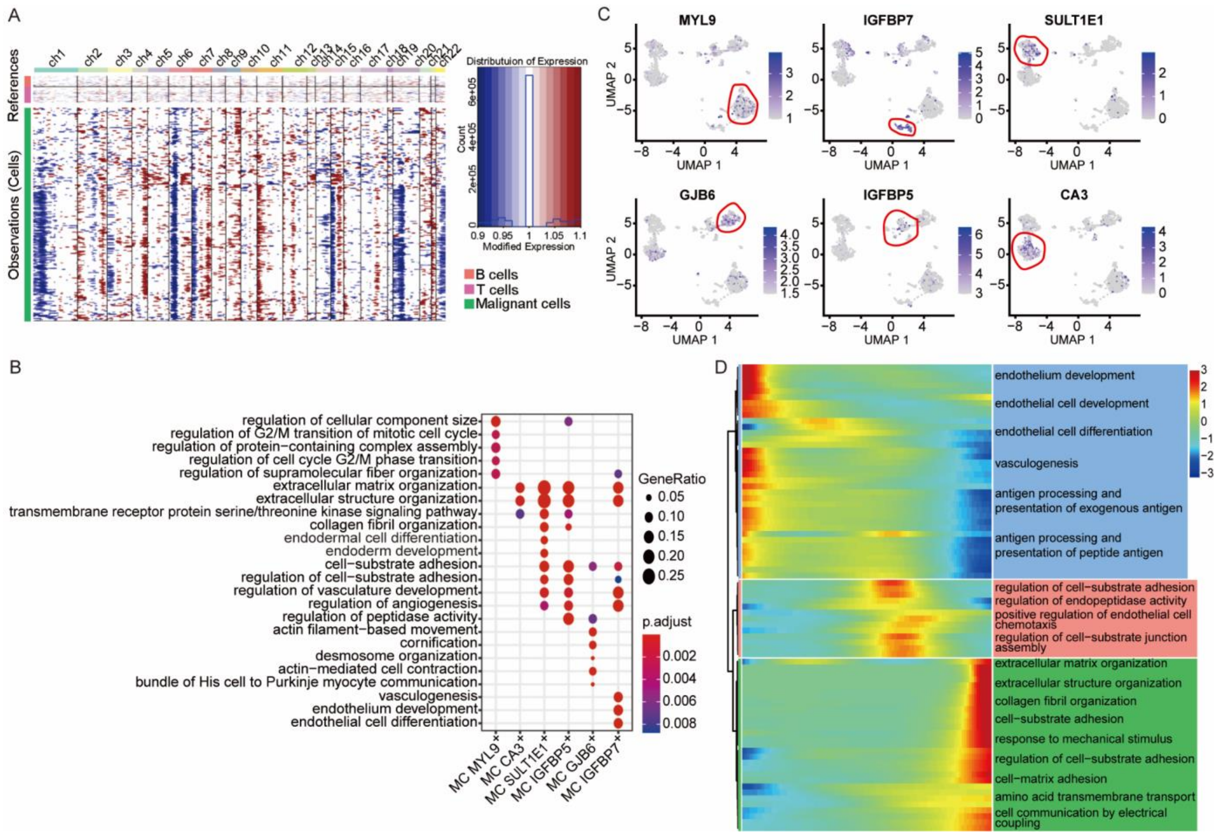

Figure S2. Gene expression features of meningioma cell (MC) subclusters

A. Heatmaps of large-scale copy number variation (CNV) events in individual cells for all three samples. B. Dot plot showing the gene ontology terms enrichment of the top 50 signature genes in different MC subclusters. C. Uniform Manifold Approximation and Projection for Dimension Reduction (UMAP) plots showing the expression of the signature markers of different MC subclusters. D. Heatmap and gene-ontology term enrichment of the top 50 signature genes of MC subclusters on a pseudotime scale.

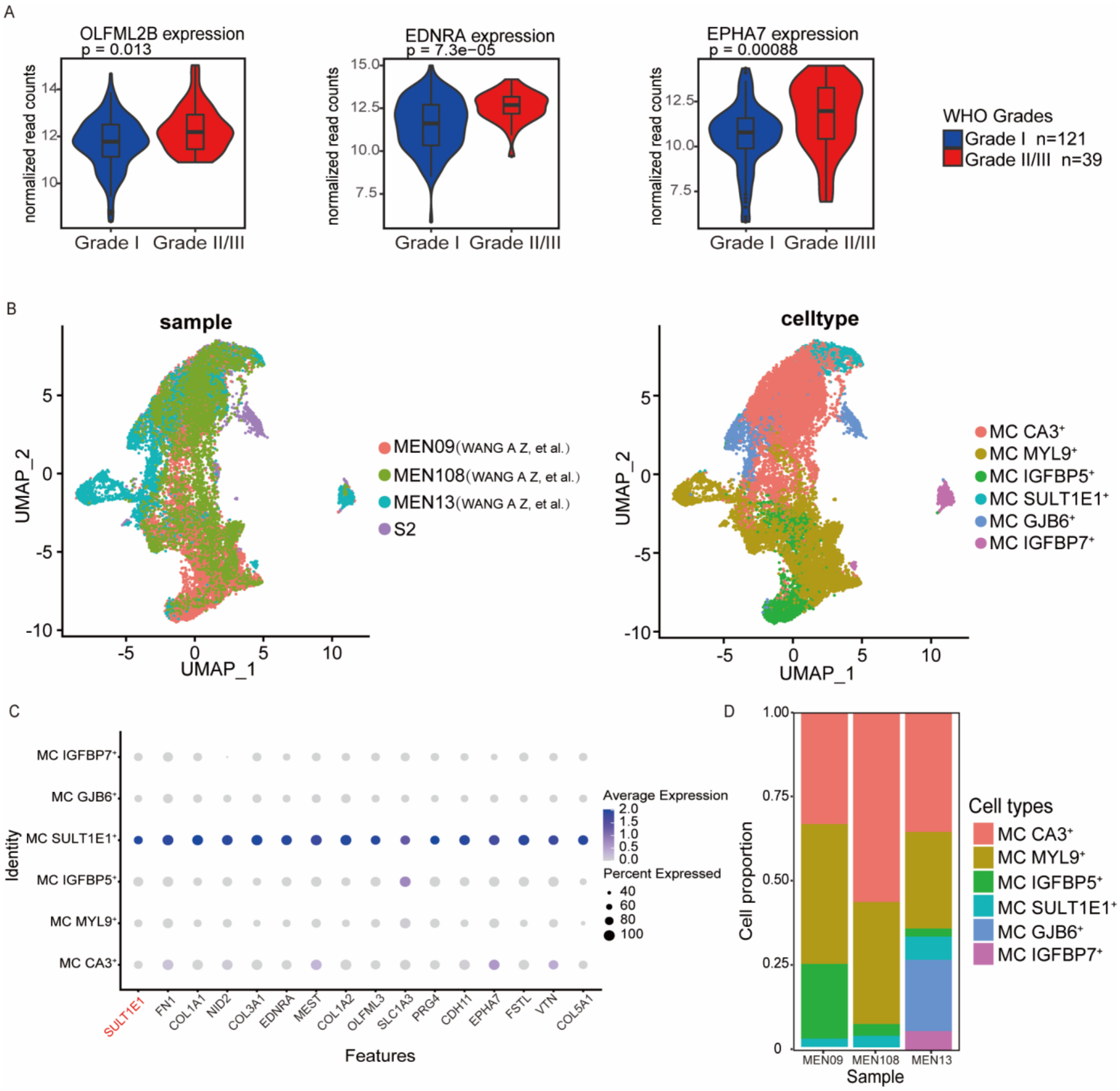

Figure S3. Validation of SULT1E1^{+} subpopulation in published data sets of high-grade meningiomas

A. Violin plot showing the expression differences of genes between grade I and grade II/III meningiomas using the Gene Expression Omnibus (GEO) dataset. B. t-SNE plots of cells across tumor samples, colored by tumor samples (left) and cell clusters (right). C. Dot plot showing the expression level of signature genes of SULT1E1^{+} subpopulation. D. Histogram indicating the proportion of cell subtypes in tumor samples.

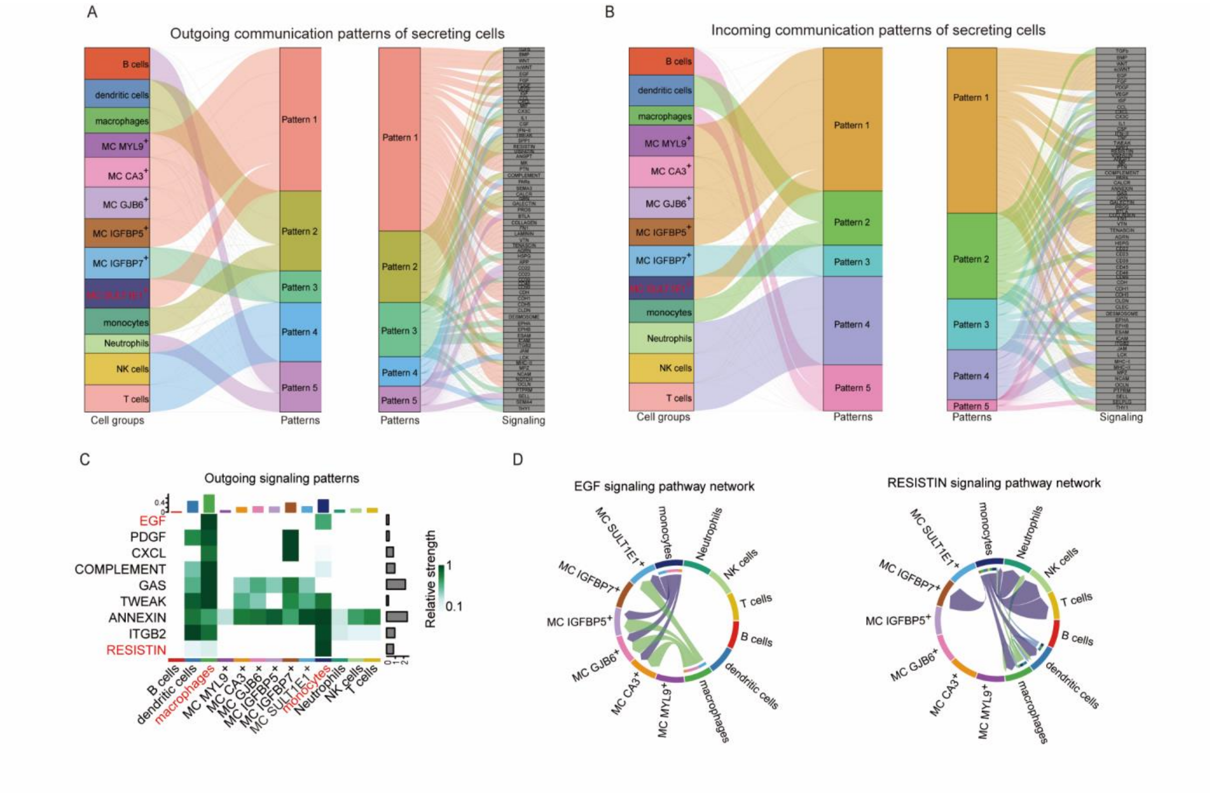

Figure S4. Cell–cell communication analysis of the MC SULT1E1^{+} subpopulation using CellChat

A. Inferred outgoing communication patterns of different cell types. B. Inferred incoming communication patterns of different cell types. C. A heatmap revealing significant outgoing signaling pathways in the macrophage/monocyte population. D. The inferred epidermal growth factor and resistin signaling networks between different cell clusters.

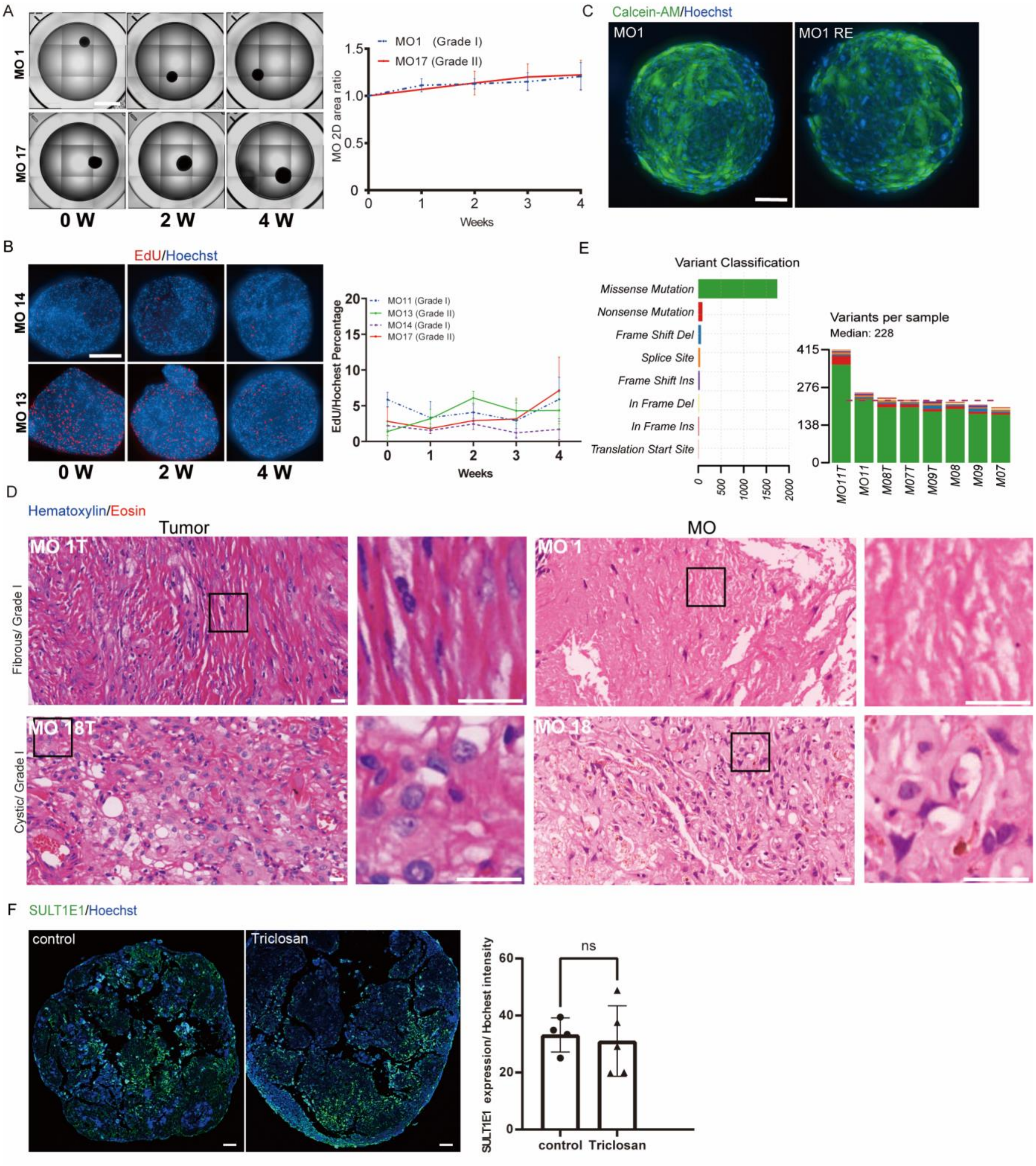

Figure S5. Characterization of meningioma organoids (MOs) after in vitro culturing

A. Representative brightfield images and quantification of the standardized 2D area ratio change of MOs; values are presented as means ± SEM. B. Representative confocal images (left) and quantification (right) of proliferating cells (EdU^+) in the MOs; scale bar: 400 µm. C. Representative confocal images of living cells (Calcein-AM^+) in continuously cultured MOs and in MOs recovered from frozen biobanks (MO RE). D. Representative hematoxylin and eosin (H&E)-stained images of parental tumors and the corresponding MOs; scale bar: 20 µm. E. Overall variant information of each sequenced sample. F. Representative confocal images

and quantification of immunostaining with anti-SULT1E1 antibody in MOs after Triclosan treatment; scale bar: 50 μ m.

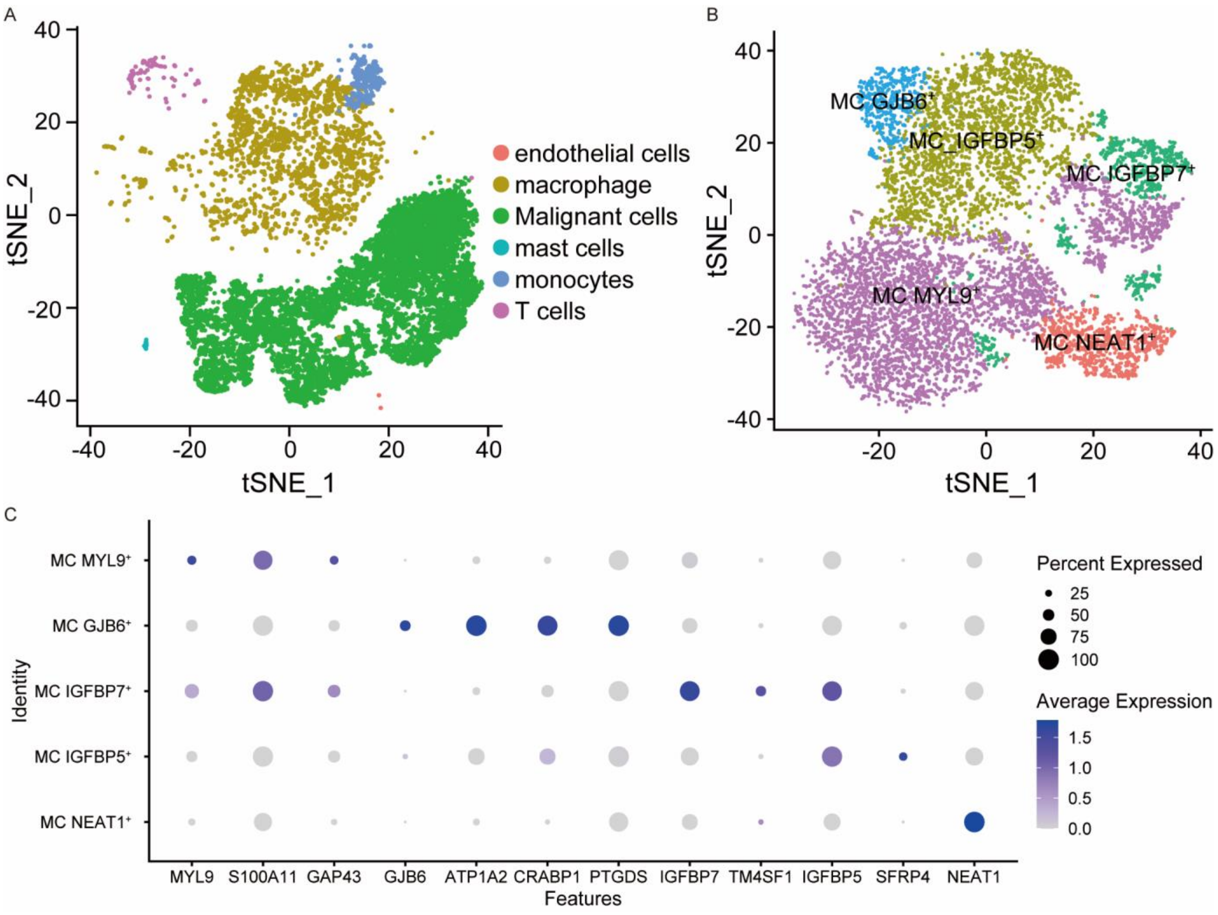

Figure S6. scRNA-Seq confirmed that MOs retained multiple meningioma cell subpopulations

A. t-SNE plots of all cells in MO sample colored by clusters. B. t-SNE plots of meningioma tumor cells in MO sample colored by subpopulation. C. Dot plot showing the expression level of marker genes of MC subpopulations identified in our study.

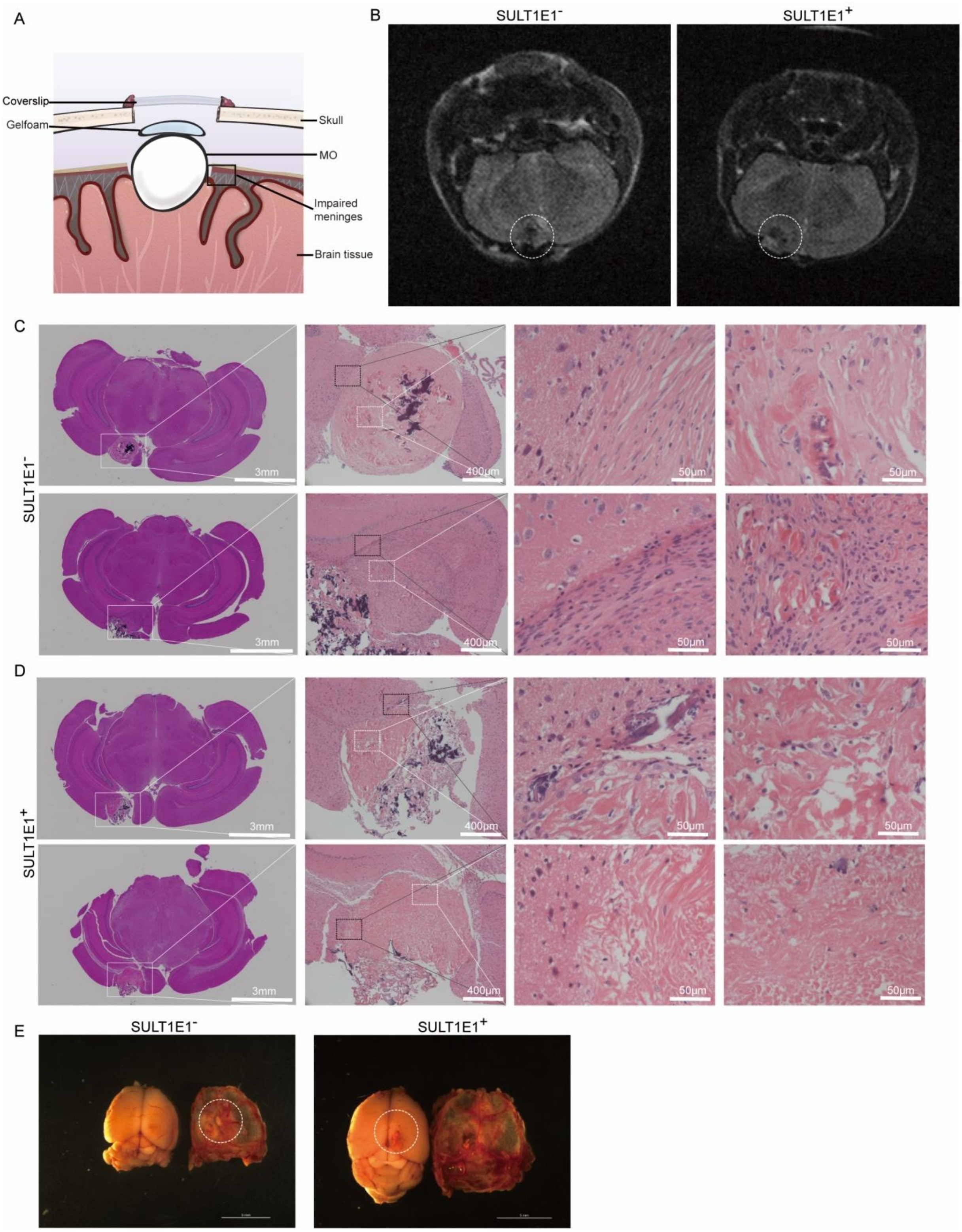

Figure S7. MOs show high survival efficacy after subdural transplantation.

A. Schematic diagram of subdural transplantation of MOs. B. Representative NMR T2-weighted (T2WI) images of mouse heads 2 months after subdural transplantation. C. Representative hematoxylin and eosin (H&E) staining of the orthotopic mouse models of subdural-transplanted SULT1E1^{-} MOs. D. Representative H&E staining of the orthotopic

mouse models of subdural-transplanted SULT1E1$^+$ MOs. E. Representative images of the dissected PDX mouse brain and skull after epidural transplantation of MOs.

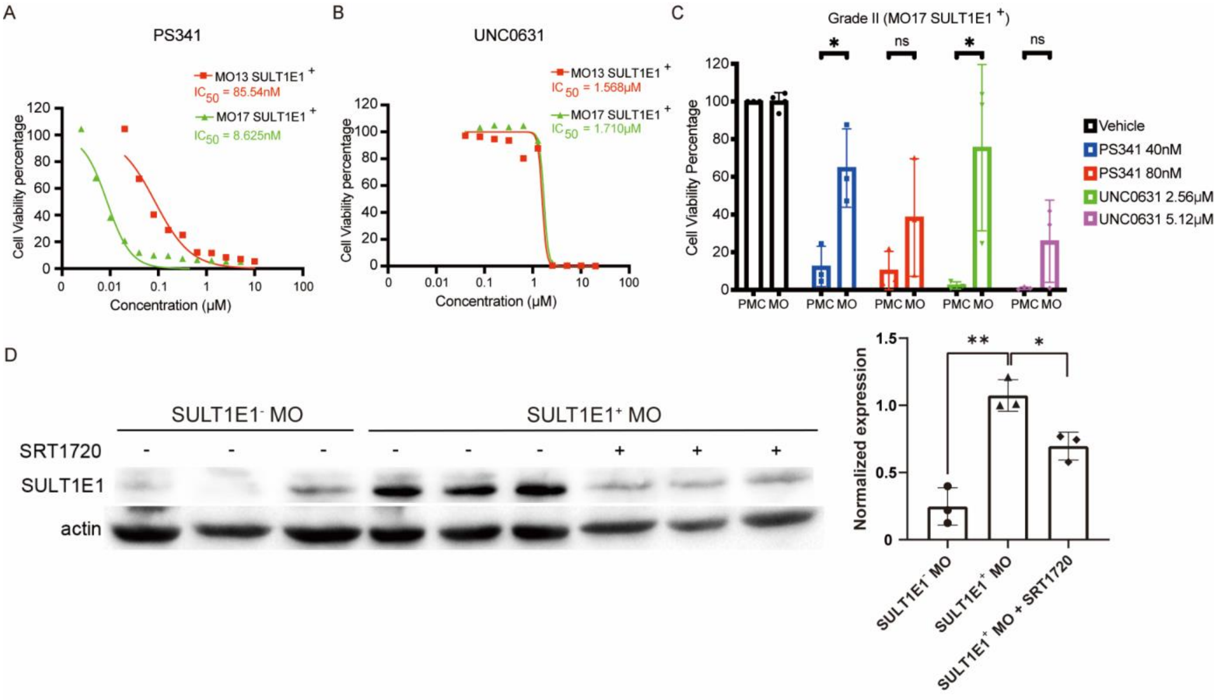

Figure S8. Inhibitory effects of PS341, UNC0631 and SRT1720 on meningioma primary cell lines and meningioma organoids (MOs)

A. Cell viability curve of PS341 for two SULT1E1$^+$ meningioma primary cell lines. B. Cell viability curve of UNC0631 for two SULT1E1$^+$ meningioma primary cell lines. C. Cell viability plots for specific concentrations of PS341 and UNC0631 for primary cell lines of meningiomas and the corresponding MOs. D. Western blotting and quantification showing significantly decrease of SULT1E1 protein expression after SRT1720 treatment in SULT1E1+ MOs.

Table 1. Clinical information of patients with established MO models.

Number	Gender	Age	Tumor Location	WHO Grade
MO1	Female	70	Convexity	I
MO4	Female	63	Convexity	I
MO7	Female	59	Convexity	II
MO8	Male	65	Convexity	I
MO9	Male	52	Convexity	II
MO10	Male	57	Skull Base	I
MO11	Female	38	Skull Base	I
MO13	Male	34	Convexity	II
MO14	Male	59	Convexity	I
MO15	Female	52	Convexity	I
MO16	Female	50	Skull Base	I
MO17	Male	42	Convexity	II
MO18	Female	66	Convexity	I
MO19	Female	61	Skull Base	I
MO20	Female	43	Skull Base	I
MO21	Female	47	Skull Base	I

MO, meningioma organoids

Table 2: Overall information of transplanted MOs.

Transplanted SULT1E1+ MOs	Epidural transplantation (Successful case#/ Brain invasion#/ Overall case#)	Subdural transplantation (Successful case#/ Brain invasion#/ Overall case#)
MO7	1/1/2	2/2/2
MO9	1/1/1	2/2/2
MO13	2/2/6	3/3/4
MO17	1/1/4	3/3/3
Brain invasion rate	100%	100%
Overall successful rate	38.5%	91%

Transplanted SULT1E1- MOs	Epidural transplantation (Successful case#/ Brain invasion#/ Overall case#)	Subdural transplantation (Successful case#/ Overall case#)
MO1	0/0/0	5/0/6
MO4	0/0/0	5/0/6
MO19	1/0/2	2/0/2
MO21	1/0/2	2/0/2
Brain invasion rate	0%	0%
Overall successful rate	50%	87.50%
